# Effect of acute resistance exercise on bone turnover in young adults before and after concurrent resistance and interval training

**DOI:** 10.14814/phy2.15906

**Published:** 2024-01-31

**Authors:** Kristen J. Koltun, Adam J. Sterczala, Nicole M. Sekel, Kellen T. Krajewski, Brian J. Martin, Mita Lovalekar, Christopher Connaboy, Shawn D. Flanagan, Sophie L. Wardle, Thomas J. O'Leary, Julie P. Greeves, Bradley C. Nindl

**Affiliations:** ^1^ Department of Sports Medicine and Nutrition, Neuromuscular Research Laboratory/Warrior Human Performance Research Center University of Pittsburgh Pittsburgh Pennsylvania USA; ^2^ Army Health and Performance Research Andover UK

**Keywords:** adaptive bone formation, bone turnover, muscle‐bone crosstalk, sex differences

## Abstract

Weight‐bearing physical activity can stimulate bone adaptation. This investigation explored the effect of an acute bout of resistance exercise before and after resistance+interval training on circulating biomarkers of bone metabolism and muscle‐bone crosstalk. Healthy young male and female participants (*n* = 21 male, 28 ± 4 years; *n* = 17 female, 27 ± 5 years) performed a 6 × 10 squat test (75% 1RM) before and after a 12‐week resistance+interval training program. Before and after completion of the training program, blood samples were collected at rest, immediately postexercise, and 2 h postexercise. Blood samples were analyzed for βCTX, P1NP, sclerostin, osteocalcin, IGF‐1, and irisin. Significant effects of acute exercise (main effect of time) were observed as increases in concentrations of IGF‐1, irisin, osteocalcin, and P1NP from rest to postexercise. A sex*time interaction indicated a greater decline in βCTX concentration from rest to 2 h postexercise and a greater increase in sclerostin concentration from rest to immediately postexercise in male compared with female participants. Sex differences (main effect of sex) were also observed for irisin and P1NP concentrations. In summary, changes in concentrations of biochemical markers of bone metabolism and muscle‐bone crosstalk were observed in males and females after an acute bout of resistance exercise and following 12 weeks of resistance+interval training.

## 1 INTRODUCTION

Habitual weight‐bearing physical activity can have important benefits for bone health across the lifespan (Kohrt et al., 2004). Exercises consisting of short‐term, intermittent, high‐impact loads are most osteogenic (Turner & Robling, 2003). However, in many active, healthy populations, bone adaptation is not the primary physiological target of exercise training, which often aims to enhance muscular strength/hypertrophy or increase aerobic fitness. For example, training to improve strength and power has emerged as a priority among military populations in preparation for performing physically demanding operational tasks (Nindl et al., 2013, 2016; Vaara et al., 2022). Notably, these same military populations are also at risk of poor bone health and bone stress injuries (Greeves et al., 2023; Koltun et al., 2022; Waterman et al., 2016). As such, it is important to understand how training programs designed to improve muscle strength and power may impact bone and bone metabolism. These data may be important for providing mechanistic insight into pathways through which physical activity can impact bone and for designing exercise training programs that target multiple physiological systems.

Structural changes in bone are a result of the balance between bone formation and resorption. Acute exercise can increase circulating biomarkers of remodeling, but effects are often variable and moderated by the type of exercise and impact loading (Dolan et al., 2022; Lester et al., 2009). Most of the literature to date suggests that acute endurance exercise, such as running and cycling, can transiently increase concentrations of bone resorption markers (i.e., βCTX) (Barry et al., 2011; Haakonssen et al., 2015; Kohrt et al., 2018, 2019; Scott et al., 2010, 2011; Sherk et al., 2017; Staab et al., 2023). Much less work has been conducted investigating the acute bone turnover responses to resistance training and with variable results reporting an increase in markers of bone resorption (Rogers et al., 2011; Whipple et al., 2004) or no change in bone turnover markers (Bemben et al., 2015; Rogers et al., 2011). Notably, resistance training can encompass a broad set of exercise types and intensities that will impact loading patterns and the skeletal stimulus, thereby potentially contributing to some of the variation among results. Additionally, chronic resistance exercise training can influence bone metabolism (Fujimura et al., 1997; Hu et al., 2011), but there are limited data regarding whether the acute bone metabolic response to resistance exercise is impacted by training status. Although assessments of bone turnover markers allow for timely monitoring of short‐term changes, there remains a need to demonstrate potential link(s) between changes in circulating biomarker concentrations to actual structural bone adaptations.

In addition to the direct effects of weight‐bearing physical activity to stimulate bone adaptation via mechanotransduction by bone cells, there can also be indirect effects via changes in the biochemical milieu. Myokines, such as insulin‐like growth factor 1 (IGF‐1) and irisin, are released following exercise and have been implicated in bone remodeling (Greeves et al., 2023; Kirk et al., 2020). Acute increases in IGF‐1 have been observed following a ballistic exercise bout in a combined sample of males and females, which was not influenced by 12 weeks of training (Sterczala et al., 2022), and in resistance trained males and females (Hatfield et al., 2021). Similarly, irisin concentrations increase following acute exercise in males, in which those with higher fitness had greater postexercise rises (Fox et al., 2018). Although exercise‐induced increases in IGF‐1 and irisin concentrations are evident, additional work is needed to relate myokines with bone adaptation.

Despite known sex differences in bone parameters between males and females, most studies examining the effects of acute resistance exercise on bone turnover have been conducted only in a single sex sample and predominantly in males (Bemben et al., 2015; Rogers et al., 2011; Whipple et al., 2004). Females, however, are at greater risk than males for bone stress injuries (Wentz et al., 2011) and osteoporosis (Salari et al., 2021). Additionally, integration of females into military combat roles following the 2013 rescindment of the Combat Exclusion Policy may serve as an impetus to participate in resistance training programs to improve performance in occupational tasks (Nindl, 2015; Nindl et al., 2016, 2017). As such, it is essential to understand how males and females might respond to a similar exercise bout as differences may help to explain sex dichotomies in musculoskeletal health and adaptations to training.

We implemented a 12‐week strength and power resistance training program with concurrent interval training (resistance + interval training) in male and female healthy young adults. The training program was designed to enhance preparedness and performance in physically demanding military operational tasks (Sterczala et al., 2023). Here, we examined the effect of an acute bout of resistance exercise on biomarkers of bone metabolism and muscle‐bone crosstalk, before and after training. Additionally, associations between the exercise‐induced biochemical response and structural bone adaptations were explored. It was hypothesized that increases in all biomarkers would be observed following the acute bout of resistance exercise, which will be influenced by sex but not by training status. Findings of this investigation may provide potential insight on exercise‐associated mechanisms of bone adaptation and help design exercise training programs.

## 2 MATERIALS AND METHODS

### 2.1 Study design

Data presented are part of the larger Soldier Performance and Readiness as Tactical Athletes (SPARTA) study supported by UK Ministry of Defence (WGCC 5.5.6‐Task 0107). Participants completed a testing battery before and after a 12‐week resistance + interval training intervention that included bone and body composition imaging and, on a separate day during the early morning, an acute resistance exercise test (ARET) and blood draws.

### 2.2 Participants

Physically active (≥30 min of physical activity ≥3 times·week^−1^) military recruit‐aged (18–36 years) male and female civilians who were free of any musculoskeletal injuries or conditions that could impair physical performance or musculoskeletal adaptations to exercise training were enrolled. Exclusion criteria were (1) currently training for a competitive sporting event; (2) body mass fluctuation ≥10 lbs within past 2 months; (3) medical condition that prevented exercise; (4) musculoskeletal injury that restricted physical activity within past 2 years; (5) current pregnancy or became pregnant during the study; (6) allergy to lidocaine; (7) medication that included anticoagulants or affected hormone concentrations (except hormonal contraceptives); (8) current asthma diagnosis; (9) history of heart condition or high blood pressure; (10) chest pain during rest, activities of daily living, or physical activity; (11) lost balance or consciousness due to dizziness within past 12 months; (12) required by treating physician to be medically supervised during physical activity; and (13) history of drug addiction or regular use of recreational drugs.

All participants provided written informed consent following an explanation of risks and benefits involved in participation. The Institutional Review Board of the University of Pittsburgh (IRB No.19030387) and United Kingdom Ministry of Defence Research Ethics Committee (903/MODREC/18) approved the study.

### 2.3 Training program

Each participant completed a 12‐week periodized concurrent resistance + interval training program (Sterczala et al., 2023). Participants completed three training sessions per week for a total of 36 training sessions. Each 60–90 min session was supervised by a National Strength and Conditioning Association Certified Strength and Conditioning Specialist and consisted of a dynamic warmup, resistance training, and interval training. The resistance training program consisted of four mesocycles: General Physical Preparedness (2 weeks), Preparation for Peak Force Production (1 week), Peak Force Development (3 weeks), and Rate of Force Development (3 weeks). A deload week followed the second, third, and fourth mesocycles to allow for adequate recovery and to prevent overtraining. During deload weeks, training intensity was reduced by 50%, one repetition maximums (RMs) were tested, and participants were familiarized with the next mesocycle's exercises. Exercise selection focused on multi‐joint, complex movements including variations of squats, deadlifts, lunges, jumps, loaded carries, presses, pullups, and Olympic lift derivatives. Interval training exercise modalities included variations of runs/sprints, upper and lower body bodyweight plyometrics, and loaded carries. Interval training during the first and second mesocycles was performed at 70%–85% of age‐predicted maximal heart rate (HRmax). For the third and fourth mesocycles, interval training intensity was performed at >80% HRmax for the first two sessions each week, while the third training session was performed at a lower intensity. Participants were instructed to abstain from excessive physical activity outside of training sessions and maintain their pre‐study dietary routine throughout the study.

### 2.4 Acute resistance exercise test

Prior to (baseline) and following (12 weeks) the 12‐week training program, each participant completed an ARET consisting of 6 sets of 10 repetitions with 75% of their 1RM squat. Each set of the ARET was separated by a 2‐min rest period. The 1RM squat was performed according to National Strength and Conditioning Association protocols. The ARET load started at 75% 1RM, but could be adjusted to ensure the participant completed all required repetitions and sets. Ratings of perceived exertion (RPE) were assessed on a 10‐point scale after each set.

### 2.5 Blood sampling and biochemical analyses

Blood samples were collected via venipuncture at rest (pre), immediately following completion of the ARET (post), and 2 h after finishing the ARET (2 h post) to assess biomarkers of bone turnover and muscle‐bone crosstalk. Blood draws were performed in the early morning following an overnight fast and no exercise for 72 h. Plasma was collected in EDTA tubes and serum samples were allowed to clot for 30 min. All vacutainers were centrifuged at 2000*g* for 15 min at 4°C with sample transferred to microtubules and stored at −80°C until analyses.

Commercially available enzyme‐linked immunosorbent assays (ELISAs) were used to determine concentrations of all analytes. Human procollagen Type 1 N‐terminal propeptide (P1NP) was analyzed using a sandwich assay (NOVUS Biologicals, NBP2‐76465, Littleton, CO; sensitivity: 9.38 pg/mL), intra‐assay and inter‐assay coefficients of variations (CVs) were 5.05% and 5.07%, respectively. Beta‐cross‐linked c‐telopeptide (βCTX) was analyzed using a sandwich assay (immunodiagnosticsystems, AC‐02F1, Gaithersburg, MD; sensitivity: 0.020 ng/mL), intra‐assay and inter‐assay CVs were 2.6% and 7.7%, respectively. Osteocalcin was analyzed using Luminex xMAP technology (Milliplex Bone Panel, HBNMAG‐51K, Millipore, Billerica, MA; sensitivity: 68.5 pg/mL), intra‐assay and inter‐assay CVs were <10% and <15%, respectively. Sclerostin was analyzed using a sandwich assay (Biomedica, BI‐20492, Germany; sensitivity: 3.2 pmol/L), intra‐assay and inter‐assay CVs were 6.0% and 6.5%, respectively. IGF‐1 was analyzed using a sandwich assay (Alpco, 22‐IGFHU‐E01, Salem, NH; sensitivity: 0.091 ng/mL), intra‐assay and inter‐assay sensitivity were 5.8% and 6.2%, respectively. Irisin was measured via competitive assay (Adipogen, AG‐45A‐0046YEK‐KI01, San Diego, CA; sensitivity: 1 ng/mL), intra‐assay and inter‐assay CVs were 6.9% and 9.1%, respectively.

### 2.6 Musculoskeletal imaging

Total body DXA scans were performed to assess body composition. Participants were scanned on a Lunar iDXA (GE Healthcare, Illinois, USA) and all scans were analyzed using enCORE Software, version 15 (GE Healthcare Lunar). High‐resolution peripheral quantitative computed tomography (HR‐pQCT) scans were performed to assess volumetric BMD (vBMD) and estimated strength of the nondominant tibia; the contralateral limb was scanned if participants had a prior fracture or metal artifact within the scan area of the nondominant limb. Scans were acquired with isotropic voxel size of 60.7 μm and energy/intensity of 68 kVp and 1470 μA. Custom 4% and 30% offset control files, representing the relative position of the center slice to the reference line were used for scan acquisition, generating 168 slices. Raw images were evaluated for motion artifacts and repeated, if necessary. Trabecular vBMD at the tibial metaphysis (4% site) and cortical vBMD at the tibial diaphysis (30% site) were assessed using 2D and 3D standard histomorphometric evaluation (XtremeCTII version 6.6 Scanco Medical AG, Brüttisellen, Switzerland). Finite element analysis (XtremeCTII version 1.13, Scanco Medical AG, Brüttisellen, Switzerland) was performed to estimate failure load (F.Ult, N) at each site. Measures of vBMD and failure load at each tibial site were selected a priori from the available HR‐pQCT measures to relate biomarkers to adaptation within specific bone sub‐compartments.

### 2.7 Statistical analyses

Data were screened and normality was assessed using the Shapiro–Wilk test. Baseline sex differences were compared via independent *t*‐tests or Mann–Whitney *U* tests. A 2*2 repeated measures ANOVA was used to assess changes in ARET load before and after the 12‐week training program and between sexes. Generalized linear mixed effects models tested the effects of exercise (main effect time: pre, post, and 2 h post), sex (main effect sex: male and female), and training status (main effect training: baseline and 12 weeks), and their interactions, on concentrations of biochemical markers of bone metabolism. For variables with a significant interaction effect, simple contrasts using sequential Bonferroni correction were performed. To explore associations among biochemical markers of bone metabolism and structural musculoskeletal outcomes, multiple linear regression comparing absolute changes in concentrations from resting to immediately postexercise at the pretraining timepoint versus the absolute change in trabecular vBMD and estimated strength at the tibial metaphysis (4% site) and cortical vBMD and estimated strength at the tibial diaphysis (30% site), while controlling for baseline values of each, were run. In cases of missing data, such as due to inadequate sample for analyses (*n* = 66, 4.8%), Little's missing completely at random (MCAR) test was performed and indicated that data were missing completely at random. No data were imputed. IBM SPSS Statistics for Windows (Version 28.0. Armonk, NY: IBM Corp.) was used for analyses. Baseline descriptive characteristics were reported as mean ± standard deviation and longitudinal outcomes reported as estimated marginal means ± standard error. Significance was set as *α* = 0.05.

## 3 RESULTS

For this investigation, 433 individuals were screened for eligibility, 247 met the inclusion criteria and 66 enrolled. During the study, 14 participants voluntarily withdrew due to personal reasons and 13 were withdrawn by the study team due to: noncompliance with study procedures (*n* = 6), injuries sustained during study procedures (e.g., low back pain, *n* = 3), injuries sustained outside of the study (*n* = 2), and the research stoppage resulting from the COVID‐19 pandemic (*n* = 2). Thus, 21 male participants and 18 female participants completed the investigation. Of the 39 participants who completed the overall study, the results presented herein include data from the 21 male and 17 female participants (*n* = 38) with bone turnover data available.

### 3.1 Primary study outcomes

We have previously reported that the 12‐week training program improved physical performance as assessed by military relevant tests (i.e., seated medicine ball throw, casualty drag, single lift, water can carry, repeated lift and carry, 2 km load carriage, and 2 km run) and maximal strength (e.g., deadlift, squat, and bench press 1RM) (Sterczala et al., 2023). Additionally, improved body composition, indicated by increased lean mass and decreased body fat, was observed following training, but on average, no changes in total bone density, microarchitecture, or strength were evident (Sekel et al., Under Review).

### 3.2 Acute resistance exercise test

Participant demographics are presented in Table 1. Data for the 6 set × 10 repetition ARET is presented in Table 2. The absolute ARET load ranged from 20–128 kg at baseline (males: 22–128 kg, females: 20–67 kg) to 16–133 kg (males: 16–133 kg, females: 20.5–73 kg) at 12 week. The average absolute load of the ARET was greater in males than females (main effect sex: *p* = 0.001) and did not change following training (main effect training: *p* = 0.127). Additionally, there were no significant effects when expressed relative to body mass.

**TABLE 1:** Baseline participant demographics.

	Men	Women	*p*‐Value
Age (years)	28.0 ± 4.2	26.9 ± 5.4	0.460
Height (cm)	178.4 ± 8.6	164.9 ± 5.9	**<0.001**
Weight (kg)	84.9 ± 14.1	64.7 ± 11.2	**<0.001**
Lean mass (kg)	59.9 ± 6.4	42.3 ± 5.8	**<0.001**
Fat mass (kg)^a^	21.8 ± 9.9	20.2 ± 7.5	0.750
Body fat (%)	24.8 ± 7.9	30.5 ± 7.0	**0.026**

^a^ Groups compared via Mann–Whitney *U*‐test; **Bold** indicates statistically significant difference between groups; Data are mean ± SD.

**TABLE 2:** Acute resistance exercise test intensity.

	Pre				Post			
	Males		Females		Males		Females	
	Weight (kg)	RPE^a^	Weight (kg)	RPE^a^	Weight (kg)	RPE^a^	Weight (kg)	RPE^a^
Set 1	73.3 ± 24.8	6 ± 2	43.3 ± 11.8	6 ± 1	75.4 ± 37.6	7 ± 2	53.9 ± 14.0	7 ± 1
Set 2	73.7 ± 24.7	8 ± 2	43.6 ± 11.4	7 ± 1	75.2 ± 37.2	8 ± 1	53.9 ± 14.0	8 ± 1
Set 3	73.9 ± 23.8	8 ± 2	44.9 ± 11.6	8 ± 1	75.0 ± 37.4	9 ± 1	53.9 ± 14.0	9 ± 1
Set 4	72.8 ± 23.1	9 ± 1	44.7 ± 11.6	9 ± 1	74.2 ± 36.8	9 ± 1	53.3 ± 13.7	9 ± 1
Set 5	69.9 ± 20.1	9 ± 1	44.5 ± 12.3	9 ± 1	72.7 ± 35.4	9 ± 1	52.8 ± 13.8	10 ± 1
Set 6	69.7 ± 20.0	10 ± 1	44.4 ± 12.5	9 ± 1	72.4 ± 34.9	10 ± 1	52.6 ± 13.7	10 ± 1

^a^ RPE scale range 0–10; Data are mean ± SD.

### 3.3 Biomarkers of bone turnover and muscle bone‐crosstalk response to acute exercise

**FIGURE 1:**
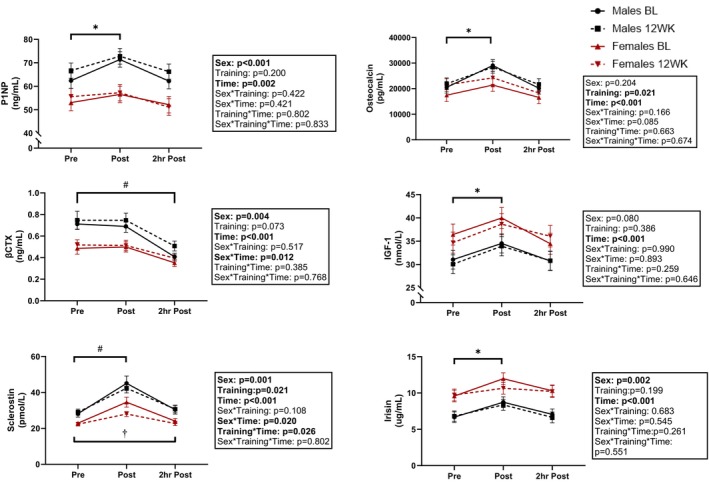
Changes in bone‐related biomarkers following acute resistance exercise in men and women prior to (BL) and following a 12‐week training intervention (12WK). *Indicates significant difference between pre‐ and postexercise (main effect time) #Indicates significant difference from Pre (sex*time interaction). †Indicates significant difference between pre and recovery, at baseline only (training*time interaction). Sample sizes vary due to inadequate sample for all analyses.

Concentrations of bone remodeling markers are presented in Figure 1. Males had significantly (20%) greater concentrations of P1NP than females when pooled across timepoints (main effect sex: *p* < 0.001), and P1NP increased by 8% from pre‐ to postexercise in the combined sample of males and females (main effect time: *p* = 0.015), before returning to resting concentrations by the 2 h post timepoint. Osteocalcin increased by 28% from pre‐ to immediately postexercise in males and females (main effect time: *p* < 0.001), but returned to preexercise values within the 2 h post time frame, and was 9% greater than baseline following 12 weeks of resistance + interval training (main effect training: *p* = 0.021). A significant sex*time interaction (*p* = 0.012) was observed for βCTX such that males had a greater decline in βCTX concentration from pre to 2 h post than females (−37% vs. −28%). Significant sex*time (*p* = 0.020) and training*time (*p* = 0.026) interactions were observed for sclerostin. The sex*time interaction indicates that both sexes had an increase in sclerostin concentrations from pre‐ to postexercise, but the increase was greater in males than females (52% vs. 38%). The training*time interaction indicates that prior to training, sclerostin remained elevated at the 2 h post timepoint, but at posttraining, sclerostin returned to baseline concentrations by 2 h post in the combined sample of males and females.

Regarding muscle‐bone crosstalk, IGF‐1 and irisin concentrations increased in the combined sample of males and females from pre‐ to immediately postexercise (main effect time: *p* < 0.001) by 13% and 24%, respectively. Concentrations were not different from pre at 2 h post (*p* ≥ 0.238). For irisin, there was also a main effect of sex (*p* = 0.002); females had significantly (34%) greater concentrations than males when pooled across all timepoints.

### 3.4 Associations among bone remodeling with structural bone outcomes

Associations among biochemical markers of bone metabolism and bone adaptation are presented in Table 3. When examining associations among exercise‐induced changes in concentrations of markers of bone remodeling and changes in trabecular (0.34 ± 2.92 mg HA/cm^3^) and cortical (−0.40 ± 9.09 mg HA/cm^3^) vBMD and estimated strength (4%: 216.29 ± 911.32 N; 30%: −33.02 ± 294.26 N), only βCTX was significant (*R* ^2^ = 0.296, F (3, 30) = 4.207, *p* = 0.013). Specifically, the change in βCTX from rest to immediately postexercise was negatively associated with change in cortical vBMD during the 12‐week training program (*β* = −0.369, *p* = 0.039), after adjusting for baseline values.

**TABLE 3:** Associations among acute changes in bone‐related biomarkers and bone adaptation during 12 weeks of exercise training.

	∆4% Tb.vBMD^a^			∆4% F.Ult^a^			∆30% Ct.vBMD^a^			∆30% F.Ult^a^		
	*β*	95% CI	*p*‐Value	*β*	95% CI	*p*‐Value	*β*	95% CI	*p*‐Value	*β*	95% CI	*p*‐Value
∆P1NP	−0.166	0, 0	0.387	−0.055	−0.031, 0.023	0.763	−0.007	−0.036, 0.971	0.971	0.289	−0.003, 0.017	0.163
∆βCTX	0.039	−7.842, 9.510	0.846	0.243	583.122, 3057.769	0.191	**−0.369**	**−47.487, −1.327**	**0.039**	0.069	−760.221, 1064.715	0.736
∆Osteocalcin	0.152	0, 0	0.431	0.077	−0.037, 0.059	0.652	0.095	0, 0.001	0.601	0.108	−0.012, 0.022	0.573
∆Sclerostin	−0.111	−0.128, 0.073	0.584	−0.033	−34.016, 28.984	0.871	−0.287	−0.521, 0.082	0.148	−0.064	−11.776, 8.546	0.748
∆Irisin	−0.112	−0.448, 0.213	0.474	−0.048	−133.642, 102.471	0.789	−0.130	−1.51, 0.645	0.419	0.148	−24.071, 56.747	0.415
∆IGF‐1	−0.062	−0.290, 0.213	0.755	0.015	−73.115, 78.847	0.939	−0.215	−1.138, 0.320	0.261	−0.088	−32.064, 20.673	0.662

*Note*: Bold indicates significant association (*p* < 0.05).

Abbreviations: CI, confidence interval; F.Ult, failure load.

^a^ All models controlled for baseline values for the independent and dependent variable.

## 4 DISCUSSION

In this investigation, transient increases in concentrations of myokines and biomarkers of bone formation and osteocyte activity, but not bone resorption, were observed following an acute bout of high load resistance exercise. The acute effects of exercise were similar between sexes, except for βCTX and sclerostin, in which males had a more exaggerated response than females. Chronic exercise, in the form of 12 weeks of concurrent resistance + interval training, was associated with favorable changes in bone turnover markers to include increased concentrations of osteocalcin and a shorter timeframe of recovery for sclerostin concentrations to return to baseline levels following exercise. Additionally, reductions in βCTX concentrations following acute resistance exercise were associated with skeletal adaptation during the 12‐week training program. Bone metabolic responses to exercise are likely specific to the training stimulus performed and this project adds to the relative dearth of studies that have examined the influence of resistance training on bone. These findings also align with the conclusions of Lester et al. (2009), which suggested that high‐volume exercise programs that incorporate resistance exercise provide a potential for inducing biochemical changes in bone metabolism. As such, high‐load resistance training may be beneficial, or at least not maladaptive, for bone outcomes while improving physical performance and, therefore, have utility for optimizing military readiness.

Concentrations of P1NP and osteocalcin increased following acute resistance exercise (main effect time). Generally, small increases in P1NP have been observed shortly after aerobic exercise cessation, but to date, insufficient data have been available to evaluate the response to resistance exercise (Dolan et al., 2022). For example, 60 min of continuous running at 55%–75% of VO_2_max (Scott et al., 2011) and of self‐paced load carriage (with 30% of body weight) (Staab et al., 2023) both increased P1NP by >10% immediately following exercise. One previous resistance training study reported no effect on bone formation, but only assessed bone alkaline phosphatase (Rogers et al., 2011). Alternatively, P1NP and osteocalcin concentrations were decreased in healthy young men and women following a bout of 4 sets of 8–10 repetitions at 80% of 1RM on a seated leg press machine (Stunes et al., 2022); however, participants were not fasted and consumed a carbohydrate energy gel prior to exercising, which may alter the bone metabolic response to exercise (Sale et al., 2015). Osteocalcin may also have exercise‐related effects beyond bone mineralization, such as glucose metabolism, that can impact concentrations (Moser & van der Eerden, 2018). Following 12 weeks of resistance + interval training, osteocalcin concentrations were increased from baseline (main effect training), which is consistent with a recent meta‐analysis that reported increases in concentrations following combined aerobic + resistance exercise training (Mohammadi et al., 2022). Results of this investigation add important information to the limited data available regarding the effect of resistance exercise on P1NP and osteocalcin.

In contrast to endurance exercise (Wherry et al., 2022), the high‐load resistance exercise bout employed in the present study was associated with decreased concentrations of βCTX 2 h postexercise (main effect time). A recent meta‐analysis suggests that exercise mode and impact level can influence circulating βCTX concentrations, with the largest postexercise increases observed following aerobic exercise and low impact/repetitive loading types, particularly those that are continuous and of long duration (Dolan et al., 2022). Alternatively, decreased concentrations have been observed in young women and men following strength training, which reached a nadir 3 h postexercise before recovering to baseline levels after 24 h (Stunes et al., 2022). The decrease in βCTX concentration in the current investigation likely reflects the typical circadian pattern, which decreases throughout the morning (Qvist et al., 2002). Decreases in βCTX concentrations throughout a morning are further supported by evidence from control trials that included a non‐exercising condition (Dolan et al., 2022), thereby suggesting that the resistance bout employed in this investigation may not stimulate an increase in βCTX beyond the typical diurnal pattern. Interestingly, exercises that do not elicit a rise in βCTX concentrations may be beneficial for skeletal tissue as the acute change in βCTX was the only variable significantly associated with structural changes in bone over a 12‐week training period. Although vBMD did not change on average in this cohort, the association may represent the prevention of maladaptation. In fact, reducing the resorption stimulus of exercise may be particularly relevant in military populations due to the high rate of bone stress injuries (Waterman et al., 2016), which can occur when targeted bone remodeling results in temporary porosity (Hughes et al., 2017, 2020).

Immediately after acute exercise, sclerostin concentrations were elevated in males and females (sex*time interaction effect), but returned to resting values within the 2 h postexercise period only after 12 weeks of training (training*time interaction effect). Postexercise increases in sclerostin are intriguing as suppression would be expected to have anabolic effects on bone adaptation. Indeed, mechanical loading reduced sclerostin gene and protein levels in a rodent model and has thereby been proposed as a mechanism through which loading can have osteogenic effects (Robling et al., 2008). In humans, however, sclerostin concentrations are often elevated following aerobic exercise (Kouvelioti et al., 2018; Pickering et al., 2017; Staab et al., 2023), but not following resistance exercise (Prowting et al., 2022; Stunes et al., 2022). Differences among results could be related to the influence of impact loading, modality, and or/intensity (Borzooeian et al., 2023; Dror et al., 2022; Kouvelioti et al., 2018). As proposed by Pickering et al. (Pickering et al., 2017), the postexercise increase in systemic levels of sclerostin could be due to changes in kidney regulation or an acute release of previously synthesized sclerostin as it is unlikely that a short bout of exercise would allow time for protein synthesis. Despite the acute increase following exercise observed in this investigation, a favorable effect of training was observed such that concentrations returned to resting levels more quickly following 12 weeks of concurrent resistance + interval training (training*time interaction effect). Although previous studies did not assess acute postexercise changes, exercise training has been associated with lower concentrations of sclerostin (Amrein et al., 2012; Hinton et al., 2017; Hughes et al., 2018), which could also be driven by changes in body composition and/or metabolism (Kurgan et al., 2022). Given its role as an osteocyte‐secreted, negative regulator of bone formation, additional work regarding the effect of exercise on sclerostin may provide important insight into how physical activity can influence bone adaptation.

In addition to assessing biomarkers of bone metabolism, the current investigation also included circulating factors released from nonskeletal tissues to explore potential interactions among organ systems in how physical activity can impact bone. Both irisin and IGF‐1 concentrations were increased immediately postexercise, which is consistent with previous work (Anastasilakis et al., 2014; Fox et al., 2018; Hatfield et al., 2021; Sterczala et al., 2022), but changes in neither IGF‐1 nor irisin were associated with structural bone adaptations. Irisin is a novel determinant of bone metabolism based on findings that irisin treatment increased cortical bone mass and strength (Colaianni et al., 2015) and prevented unloading‐induced bone loss in male mice (Colaianni et al., 2017), and stimulated osteoblast differentiation in vitro (Colaianni et al., 2014). Factors associated with muscle‐bone crosstalk likely have an important role in how physical activity impacts bone adaptation, but much more work is needed to better understand interactions among components of the musculoskeletal system.

Males, on average, have greater bone mineral content, area, and areal bone mineral density than females (Nieves et al., 2005), but few studies have examined sex differences in the acute bone‐related biomarker response to exercise. In the current investigation, P1NP concentrations changed similarly between males and females following acute resistance exercise, but overall, males had greater concentrations of P1NP than females, which is consistent with previous reports from healthy military‐aged populations (Evans et al., 2008; O'Leary et al., 2022) and may partially be due to sex differences in bone size (Henry & Eastell, 2000). Irisin concentrations were affected similarly by exercise in males and females, but were higher in females than males. Resting irisin concentrations have been reported as higher in young females and males compared to adults (Al‐Daghri et al., 2014; Anastasilakis et al., 2014). Following exercise, βCTX concentrations decreased in both sexes, but to a greater extent in males. Although the circadian variation in βCTX is independent of sex (Qvist et al., 2002), healthy female adults tend to have lower concentrations than males (Evans et al., 2008; O'Leary et al., 2022), which may attenuate the magnitude of change possible. Sclerostin was also impacted to a greater extent following exercise in male participants compared to female participants (sex*time interaction effect), which could be related to males having higher sclerostin concentrations than females and/or its potential association with bone density and body composition (Amrein et al., 2012), which may differ between sexes.

Strengths of this investigation include a longitudinal design that allowed for biomarkers to be assessed around an exercise bout, both prior to and following a 12‐week exercise training program. The sample also included males and females performing the same relative exercise bout (75% 1RM) to assess for potential sex differences in the acute effect of exercise on bone‐related biomarkers. HR‐pQCT data were also incorporated to explore associations among acute changes in biomarkers and tissue level adaptation. The inclusion of several biochemical markers of bone metabolism relating to bone formation, bone resorption, osteocyte activity, and the muscle secretome also allows for an examination of the multiple pathways through which physical activity may influence bone. Limitations of the current investigation include a relatively small sample size, although robust statistical methods were implemented to utilize all data available. Additionally, as this was a secondary analysis of data from a larger study, the methodology did not include a non‐exercising control group for comparison, assessments of plasma volume or nutrition, or standardize testing based on menstrual phase or contraceptive use in women. Despite these limitations, the data presented contribute important preliminary information to the area of bone turnover responses to acute resistance exercise in males and females.

## 5 CONCLUSION

In this investigation, acute resistance exercise was associated with transient increases in concentrations of P1NP, osteocalcin, sclerostin, IGF‐1 and irisin, but not βCTX, and chronic resistance + interval training was associated with increased concentrations of osteocalcin and a shorter recovery period for sclerostin concentrations to return to baseline levels following exercise. These findings suggest that high relative load intensity resistance exercise, which may be implemented to improve strength and physical performance of military tasks, also has the potential for inducing biochemical changes in bone metabolism.

## FUNDING INFORMATION

This study was funded by the UK Ministry of Defence WGCC 5.5.6‐Task 0107.

## CONFLICT OF INTEREST STATEMENT

No conflicts of interest, financial or otherwise, are declared by the authors.

## ETHICS STATEMENT

All participants provided written informed consent following an explanation of risks and benefits involved in participation. The Institutional Review Board of the University of Pittsburgh (IRB No. 19030387) and United Kingdom Ministry of Defence Research Ethics Committee (903/MODREC/18) approved the study.

## Data Availability

Data will be made available upon reasonable request.
